# Children’s Filial Piety and Parents’ Depressive Symptoms: Findings From a Dyadic Study

**DOI:** 10.1093/geroni/igab046.766

**Published:** 2021-12-17

**Authors:** Stephanie Bergren, Qun Le, XinQi Dong

**Affiliations:** 1 Rutgers University, New Brunswick, New Jersey, United States; 2 Rutgers University, Rutgers Institute for Health, New Jersey, United States

## Abstract

Filial piety is an important Chinese cultural value that prescribes child behavior towards their parents, but little is known about its relationship to the parents’ psychological wellbeing. This study utilizes dyad data from the PINE and PIETY Studies. Filial piety was measured by asking how much the participant thought children should 1) care; 2) respect; 3) greet; 4) please and make happy; 5) obey; and 6) provide financial support to their parents. Depressive symptoms were measured by Patient Health Questionaire-9 with a cutoff of 5 indicating the presence of depressive symptoms. Logistic regressions were used to examine the associations controlling for both children’s and parents’ sociodemographic characteristics. Higher filial piety in happy (OR:0.80, (0.65-0.99)) or obey (OR:0.83, (0.68-1.00)) was associated with a lower likelihood of depressive symptoms among parents. Future research should explore the potential causal relationships between children’s filial piety and parents’ mental health.

